# *Fonsecaea* *pedrosoi* Conidia and Hyphae Activate Neutrophils Distinctly: Requirement of TLR-2 and TLR-4 in Neutrophil Effector Functions

**DOI:** 10.3389/fimmu.2020.540064

**Published:** 2020-10-21

**Authors:** Leandro Carvalho Dantas Breda, Cristiane Naffah de Souza Breda, José Roberto Fogaça de Almeida, Larissa Neves Monteiro Paulo, Grasielle Pereira Jannuzzi, Isabela de Godoy Menezes, Renata Chaves Albuquerque, Niels Olsen Saraiva Câmara, Karen Spadari Ferreira, Sandro Rogério de Almeida

**Affiliations:** ^1^ Departamento de Imunologia, Instituto de Ciências Biomédicas, Universidade de São Paulo, São Paulo, Brazil; ^2^ Departamento de Análises Clínicas e Toxicológicas, Faculdade de Ciências Farmacêuticas, Universidade de São Paulo, São Paulo, Brazil; ^3^ Departamento de Ciências Farmacêuticas, Instituto de Ciências Naturais, Universidade Federal de São Paulo, Diadema, Brazil

**Keywords:** NETs (neutrophil extracellular traps), phagocytosis, neutrophils, TLR (Toll-like receptors), migration, fungal infection, innate immunity, chromoblastomycosis

## Abstract

Chromoblastomycosis is a chronic and progressive subcutaneous mycosis caused mainly by the fungus *Fonsecaea pedrosoi*. The infection is characterized by erythematous papules and histological sections demonstrating an external layer of fibrous tissue and an internal layer of thick granulomatous inflammatory tissue containing mainly macrophages and neutrophils. Several groups are studying the roles of the innate and adaptive immune systems in *F. pedrosoi* infection; however, few studies have focused on the role of neutrophils in this infection. In the current study, we verify the importance of murine neutrophils in the killing of *F. pedrosoi* conidia and hyphae. We demonstrate that phagocytosis and reactive oxygen species during infection with conidia are TLR-2– and TLR-4–dependent and are essential for conidial killing. Meanwhile, hyphal killing occurs by NET formation in a TLR-2–, TLR-4–, and ROS-independent manner. In vivo experiments show that TLR-2 and TLR-4 are also important in chromoblastomycosis infection. TLR-2KO and TLR-4KO animals had lower levels of CCL3 and CXCL1 chemokines and impaired neutrophil migration to the infected site. These animals also had higher fungal loads during infection with *F. pedrosoi* conidia, confirming that TLR-2 and TLR-4 are essential receptors for *F. pedrosoi* recognition and immune system activation. Therefore, this study demonstrates for the first time that neutrophil activation during *F. pedrosoi* is conidial or hyphal-specific with TLR-2 and TLR-4 being essential during conidial infection but unnecessary for hyphal killing by neutrophils.

## Introduction

Chromoblastomycosis (CBM) is a chronic, progressive subcutaneous mycosis caused by different fungal species of the *Herpotrichiellaceae* family, such as *Phialophora verrucosa*, *Cladophialophora carrionii*, *Rhinocladiella aquaspersa*, *Exophiala spinifera*, *Aureobasidium pullulans*, *Chaetomium funicola*, *Fonsecaea monophora*, *Fonsecaea nubica*, and *Fonsecaea pugnacious*, but mainly by *Fonsecaea pedrosoi* (1, 2). The disease has been diagnosed on all 5 continents, but it is mainly founded in tropical and subtropical countries (3), such as Brazil (4, 5), Mexico (6), China (7), and Madagascar (8). It affects mostly farm workers because the natural habitat of this fungi is in the soil and decaying plants (9). The treatment is difficult and involves the combination of antifungal prescriptions (10), cryo/heat therapy (11), and in some cases, surgery to remove all the infected tissue (12). CBM is one of the most difficult deep mycoses to treat and has low rates of cure (13, 14). The treatment is long and expensive, and because the disease affects mainly low-income individuals, there is a high rate of treatment dropout, leading to a high rate of disease relapse (14). Therefore, a better understanding of the pathogen–host interaction is needed to improve the treatment of CBM to increase the rate of successful treatment and decrease the time and cost of treatment. It is well established in the literature that T-cells and IFN-γ are important for disease control (15–17), but little is known about the innate immune response in CBM. De Souza’s lab show that *F. pedrosoi* conidia ingested by resident macrophages are able to grow into hyphae, leading to macrophage death (18). However, IFN-γ preactivated macrophages have a fungistatic activity, decreasing hyphal growth and remaining alive (19). Neutrophils are another type of important innate immune cells during an infection process. They are the most abundant leukocytes in the bloodstream and the first cells to migrate toward the infection site (20). Neutrophils are directly responsible for pathogen killing, mainly through three different effector functions: 1) phagocytosis, 2) degranulation, and 3) neutrophil extracellular trap (NET) release. These cells can also indirectly control an infection by secreting IL-17 that attracts Th17 lymphocytes, which are an important cell population for fungal infection control (21, 22). Neutrophils can also modulate macrophage phenotypes, helping the immune system against the infection (23). However, although neutrophil activation is usually associated with pathogen containment and elimination, overactivation may be harmful to the host (24, 25), so a tight regulatory system for neutrophil activation is important (26). Although neutrophils are known to be important in several fungal infections, such as *Candida albicans* (27), *Aspergillus fumigatus* (28), *Cryptococcus neoformans* (29), *Paracoccidioides brasiliensis* (30), and *Sporothrix schenkii* (31), few studies focus on the neutrophil response during CBM infection. However, besides being found in CBM skin lesions, the significance of neutrophils in helping the immune system avoid fungal spread and promote fungal killing in CBM is unknown (32, 33). Rozental and colleagues demonstrate that the neutrophil phagocytose conidia produces reactive oxygen species (ROS) to kill the ingested conidia (34). However, which receptors are responsible for neutrophil activation and whether these cells are able to recognize and kill *F. pedrosoi* hyphal structures is still unknown. In this study, we demonstrate for the first time that conidial killing by neutrophils is TLR-2– and TLR-4–dependent. We also show that neutrophils’ hyphal killing occurs by NET release in a TLR-2–, TLR-4–, and ROS-independent manner. Taken together, our findings help to better understand the neutrophil response in the control of CBM disease caused by conidial or hyphal infection.

## Materials and Methods 

### Research Ethics Board Approval

The protocol for animal studies was approved by the ethics committee (Comissão de Ética no Uso de Animais da Faculdade de Ciências Farmacêuticas da Universidade de São Paulo) under protocol number 474. The study was conducted in accordance with Conselho Nacional de Controle de Experimentação Animal (CONCEA) and the Sociedade Brasileira de Ciências em Animais de Laboratório (SBCAL) guidelines.

### Fungal Strain and Growing Conditions 

The *F. pedrosoi* strain (CBS 271.37) was cultivated on Sabouraud agar at 30°C until the inoculum preparation. The fungi were transferred from the Sabouraud agar tube to 150 mL of potato dextrose broth (Difco, BD) and grown for 5 days at 30°C with shaking. After the growth period, the inoculum was filtered through a 40-μM cell strainer. The conidial particles were obtained from the flow-through solution while the hyphae were retained in the cell strainer (hyphae size used in this study is larger than 40 μM). Conidia-enriched samples were centrifuged for 5 min in 300x*g* to collect the remains of small hyphae and large conidia. The supernatant was collected and centrifuged for 10 min at 9000x*g* and then resuspended in 1x PBS. The concentration of conidia and hyphae was determined by Neubauer chamber counting.

### Mouse Bone-Marrow Neutrophil Enrichment 

Wild-type, TLR-2KO and TLR-4KO C57BL/6 animals at 8–12 weeks of life were used in this study. The animals were euthanized with an overdose of anesthetics according to animal ethics committee approval. Femurs and tibias were taken, and the bone marrow was collected using fetal bovine serum-free RPMI medium. The cells were passed through a cell strainer to retain the small debris and clots. The cells were washed once in 1x PBS, and neutrophil enrichment was performed using a Ficoll density layer (1119 and 1077 density) or by positive selection with anti-Ly6G magnetic beads (according to the manufacturer’s instructions; Miltenyi^®^). After neutrophil enrichment, the cells were counted using a Neubauer chamber with trypan blue staining to calculate cell viability. Sample purity was analyzed by flow cytometry (anti-Ly6G) or the cytospin technique followed by methylene blue and eosin staining (**Supplementary Figure 1**). The viability and purity of the cells used in this work exceeded 95% and 85%, respectively

### Neutrophil Fungicidal Assay

Purified neutrophils (1x10^5^) were infected with *F. pedrosoi* conidia (multiplicity of infection (MOI) 2:1; 2 fungi to 1 cell) or hyphae (MOI 1:1) for 2 h at 37°C under homogenization. Different MOIs were previously tested with similar results. Therefore, MOIs of 1:2 and 1:1 were chosen for conidia and hyphae experiments, respectively. As a control, conidia or hyphae were incubated under the same conditions without neutrophils. After incubation, an aliquot was taken and diluted in distilled water to induce neutrophil lysis without harming the fungi. The fungi were seeded onto Sabouraud agar and incubated for 5 days at 37°C for colony-forming unit (CFU) counting. The CFUs of the control groups (fungi without neutrophils) were set as 100% CFU (100% survival). To confirm whether the conidial and hyphal killing was due to phagocytosis or NET release, we first incubated the neutrophils with cytochalasin D (5 μg/mL or DMSO as a vehicle) or DNase (25 U/mL) for 15 min. The neutrophils were then lysed with distilled water, and the fungi were seeded onto Sabouraud agar. Next, to demonstrated that ROS is essential to conidia but not hyphae killing, a survival assay was performed as described above in the presence of diphenyleneiodonium (DPI), an inhibitor of NADPH-oxidase (0–20 μM).

### Phagocytosis and NET Assay

A phagocytosis assay was performed with neutrophils purified from WT, TLR-2KO, and TLR-4KO (1.5x10^5^) mice and seeded onto round coverslips previously treated with poly-L-lysine (Sigma-Aldrich^®^) and placed at the bottom of 24-well plates. Afterward, conidia (MOI 2:1) or hyphae (MOI 1:10) were added, and the plates were quickly centrifuged to increase the cell adhesion on the coverslip. After 2 h, the supernatant was removed, and the cells were fixed with 4% paraformaldehyde (PFA) for 15 min. After washing with PBS, the cells were permeabilized with 0.1% PBS-T for 15 min, and the DNA was stained with sytox green (4 μM) for 30 min and then washed with PBS. Afterward, the cells were washed, and the coverslip was placed over a slide with 5 μL of Vecta-Shield^®^ and sealed with nail polish. The slides were kept in the dark at 4°C until analysis by immunofluorescence microscopy. The phagocytic index was calculated using the following equation: (number of conidia inside the cells x 100)/ total number of neutrophils. The phagocytic index calculated in WT animals was set as 1, and the TLR-2KO and TLR-4KO phagocytic indexes were then compared to the WT. A NET assay was performed on coverslips (for immunofluorescence microscopy) or in a 96-well plate (plate reader assay). For microscopy assay, two similar protocols were used to detect NET release: single staining (sytox green for nucleic acid staining) or double staining (sytox green and antihistone staining). Briefly, cells were placed onto coverslips and infected with conidia or hyphae of *F. pedrosoi* (as described above). For single staining, after washing, fixing, and permeabilizing, cells were stained with sytox green (4 μM) for 30 min, and the slides were assembled using 5 μL of Vecta-Shield^®^. For double staining, after the permeabilization step, cells were preincubated with mouse antihistone H3 (abcam^®^) for 1 h. After washing with PBS, cells were incubated with donkey antimouse IgG conjugated with Alexa-Fluor 647 (Abcam^®^) and sytox green for 45 min. After washing, the slides were assembling with 5 μL of Vecta-Shield^®^. For NET quantification, we performed a plate reader assay where 0.5x10^5^ neutrophils (WT, TLR-2KO, or TLR-4KO) in RPMI medium were seeded into 96-well plates in the presence of sytox green (4 μM). The cells were stimulated with conidia (MOI 2:1), hyphae (MOI 1:10), or medium only (negative control) and kept at 37°C with 5% (v/v) CO_2_ incubator, and sytox green fluorescence intensities were detected by a SpectraMax M2 fluorescence microplate reader (Molecular Devices) every 30 min for 180 min. The NETotic ratio was calculated based on the value of NET formation in unstimulated neutrophils at each specific time point (ratio of 1).

### ROS Detection Assay

ROS production (O_2_^-^, H_2_O_2_, and HOCl) is usually associated with phagocyted pathogen killing. A luminol-enhanced chemiluminescence assay was used to measure the total ROS production (intracellular and extracellular ROS) during conidia and hyphae infection. Briefly, 1x10^5^ neutrophils were seeded (RPMI media) into a white 96-well plate (Costar 3917) in the presence of the luminol reagent (1 mmol/L; Sigma-Aldrich). Conidia (MOI 2:1) or hyphae (MOI 1:1) were added, and the chemiluminescence was detected with a microplate luminometer reader (EG&G Berthold LB96V, Bad Wildbad, Germany) every 2 min for 90 min. To measure the ROS production in resting neutrophils, the cells were incubated without any stimuli. The area under the curve was calculated to measure the total ROS production after the 90 min stimulation period. To compare ROS production between WT, TLR-2KO, and TLR-4KO, an unstimulated neutrophil sample of each group was set as a ratio of 1. The total ROS production of each group after conidia or hyphae stimulation was compared to its unstimulated sample. To analyze whether hyphae blocked ROS production, neutrophils were stimulated with phorbol 12-myristate 13-acetate (PMA; 100 nM), a well-known NADPH-oxidase agonist, in the presence of hyphae or heat-killed hyphae (HK-hyphae). HK-hyphae were obtained by heating hyphae for 120 min in a 90°C in a dry bath block. After heat killing, an aliquot was seeded onto Sabouraud agar plates to confirm that the hyphae were dead (data not shown).

### Chemiotaxis Assay

To verify whether the TLR-2 and TLR-4 were essential for neutrophil migration to the infection site, WT, TLR-2KO, and TLR-4KO animals were intraperitoneally (i.p.) infected with 5x10^7^ conidia or 4x10^6^ hyphae of *F. pedrosoi* (final volume of 200 μl). Animals infected with 1x PBS were used as a control. After 3 h, the animals were euthanized and the i.p. lavage was performed with 5 mL of 0.05% PBS-FBS with 2 mM EDTA to prevent clots. The cells were spun down, and the supernatant was collected to further measurement of neutrophils’ chemoattractant CXCL1 (C-X-C motif ligand 1 also known as keratinocyte-derived cytokine; KC) and CCL3 (C-C motif chemokine ligand 3 also known as macrophage inflammatory protein-1α; MIP-1α). The cells were washed and resuspended in 1x PBS and subjected to Neubauer’s chamber counting, followed by flow cytometry staining using anti-CD45, anti-CD11b, and anti-Ly6G antibodies.

### *In Vivo* Infection

To confirm that TLR-2 and TLR-4 are important receptors in CBM infection, we i.p. infected WT, TLR-2KO, and TLR-4KO animals with 5x10^7^ of *F. pedrosoi* conidia. After 24 h, the animals were euthanized, and the spleen and liver were collected to analyze the cell populations and fungal load. Briefly, the organs were harvested and smashed through a cell strainer (70 μM). An aliquot of the organ macerate was collected and seeded onto Sabouraud agar for further CFU analysis, and the rest of the organ macerate was centrifuged and placed over 3 mL of Ficoll (1119 density) to isolate the leukocytes from the other tissue cells. The leukocytes were collected from the top of the Ficoll layer and stained with anti-CD45^+^ and anti-Ly-6G^+^ for neutrophil analysis. To verify whether neutrophil populations were similar among the WT, TLR-2KO, and TLR-4KO in the basal conditions (noninfected animals) the peripheral blood, peritoneal lavage, spleen, and liver were collected, and cells were stained as described above.

### Statistical Analyses

Statistical analysis was performed using the Graphpad Prism® program. For analyses between the groups of the evaluated parameters, the following tests were applied: Student *t*-test for analyses between two groups with one variable; one-way ANOVA and post-Bonferroni test for analyses between more than two groups with one variable; and two-way ANOVA and Bonferroni posttest for group analyses with two or more variables and the Bonferroni posttest. Data are expressed as mean ± SEM, and the observed differences were considered significant when *p* < 0.05 (5%).

## Results

### Neutrophil Fungicidal Activity Against *F. pedrosoi* Conidia and Hyphae

**Figure 1:**
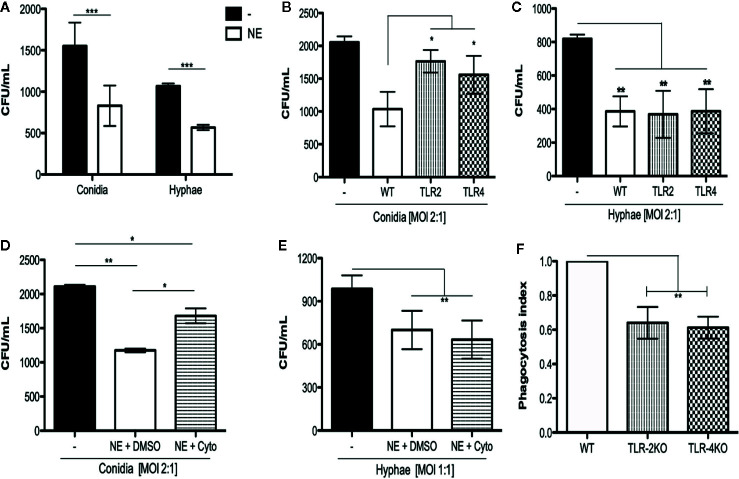
Neutrophil toll like receptors 2 and 4 are important to kill conidia but not hyphae of *F. pedrosoi*. **(A)** Neutrophils from WT were purified using magnetic beads and incubated with conidia (MOI 2:1) or hyphae (MOI 1:1) for 2 h at 37°C. Afterward, cells were lysed with sterile distilled water and seeded onto Sabouraud agar. After seeding, plates were kept at 37°C for 5 days prior to the final colony counting. As control, conidia and hyphae were incubated in the same conditions without neutrophils. Data are expressed as mean ± SEM; *n* = 5, two-way ANOVA with Bonferroni’s posttest ***p < 0.001). Using neutrophils from TLR-2KO and TLR-4KO animals, we verified that these receptors are important against conidia **(B)** but not hyphae **(C)** killing. Data are expressed as mean ± SEM; *n* = 9, one-way ANOVA with Bonferroni’s posttest. *p < 0.05; **p < 0.01; ***p < 0.001). Preincubating WT neutrophils with cytochalasin D (or DMSO – vehicle) for 30 min, we verified that phagocytosis is essential to kill *F*. *pedrosoi* conidia **(D)** but not hyphae **(E)**. Data are expressed as mean ± SEM; *n* = 5, two-way ANOVA with Bonferroni’s posttest **p < 0.01; ***p < 0.001). The importance of TLR-2 and TLR-4 in conidial phagocytosis was verified by immunofluorescence. Neutrophils were seeded over a coverslip and infected with conidia (MOI 2:1) for 2 (h) After, the cells were fixed and permeabilized and the nuclei were stained with sytox green. The slides were mounted with Vecta-Shield^®^ and sealed with nail polish. At least 100 cells were analyzed to calculate the phagocytosis. Phagocytosis index observed in WT animals were set as 1 (100%) and the phagocytosis observed in TLR-2KO and TLR-4KO animals were compared to WT phagocytic index **(F)**. Data are expressed as mean ± SEM; *n* = 4, one-way ANOVA with Bonferroni’s posttest **p < 0.01.

To determine whether neutrophils were capable of killing *F. pedrosoi* conidia and hyphae, we incubated the fungal particles with (or without) WT purified neutrophils for 2 h. This experiment was performed in microtubes because we wanted to verify the total neutrophil killing capacity, which includes not only phagocytosis, but also NET release and degranulation. After incubation, an aliquot was taken directly from the tube (with no centrifugation step) and diluted in distilled water to induce neutrophil lysis without harming the fungi. The fungi were seeded onto Sabouraud agar for 5 days for CFU counting. Conidia and hyphae incubated without neutrophils were used as control of fungal maximum growth (or no fungal killing). The CFU counts showed that, in 2 h, purified neutrophils were able to kill both conidia and hyphae (**Figure 1A**). We next questioned whether this conidial and hyphal fungicidal activity was TLR-2– and TLR-4–dependent. To answer that question, we repeated this experiment using WT, TLR-2KO, and TLR-4KO neutrophils. Our data show that the neutrophil killing activity of conidia was impaired in TLR-2KO and TLR-4KO cells (**Figure 1B**) although hyphal killing was not impacted (**Figure 1C**). These results suggest that *F. pedrosoi* particles activates neutrophils by distinct pathways.

### Phagocytosis Is a TLR-2– and TLR-4–Dependent Mechanism And Is Important for Conidial but Not Hyphal Killing

Our first hypothesis was that conidia were being killed via phagocytosis although hyphae were not able to be internalized because of their size. Therefore, we performed a killing assay using cytochalasin D, a drug well known to inhibit actin and myosin polymerization, thus inhibiting the phagocytosis process. Purified WT neutrophils were first incubated with cytochalasin D (or DMSO as a vehicle control) for 15 min and then incubated for 2 h with (or without) conidia or hyphae. Conidia and hyphae incubated without neutrophils were used to set the CFU as 100% (100% survival). We demonstrate that the phagocytosis process was responsible, at least in part, for conidial (**Figure 1D**) but not hyphal killing (**Figure 1E**). To check whether TLR-2 and TLR-4 were important for phagocytic activity, purified neutrophils from WT, TLR-2KO, and TLR-4KO animals were used to perform an immunofluorescence assay to quantify the phagocytic index of these cells. First, neutrophils were seeded onto a coverslip and incubated for 2 h with conidia (MOI 1:4). Afterward, cells were fixed and permeabilized, the nuclei were stained with sytox green, and the phagocytosis index was analyzed by counting 100 cells per group. The phagocytosis index obtained for WT neutrophils was set to a ratio of 1 (or the 100% phagocytosis index). The TLR-2KO and TLR-4KO phagocytosis indexes were calculated and then compared to the WT index. Our results show that conidia phagocytosis is impaired to approximately 35% to 45% in TLR-2KO and TLR-4KO neutrophils compared to WT neutrophils (**Figure 1F** and **Supplementary Figure 2**).

### *F. pedrosoi* Conidia Stimulate and Hyphae Block Neutrophil ROS Production

**Figure 2:**
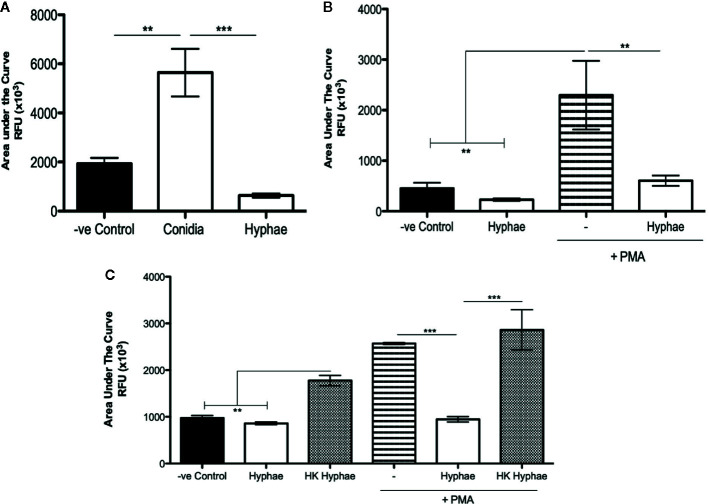
Neutrophil ROS production is stimulated by *F. pedrosoi* conidia and blocked by *F. pedrosoi* hyphae. **(A)** WT neutrophils were purified using Ficoll density layer and then seeded in a 96-well plate in the presence of a luminol reagent and stimulated with *F. pedrosoi* conidia or hyphae. The ROS production was measured every 2 min to approximately 60 min. As unstimulated control, neutrophils were incubated in the absence of fungi to measure the ROS production during the steady state. The area under the curve was calculated to measure the total ROS production after 60 min. **(B)** To confirm that hyphae block ROS production, we stimulated the cells with PMA (highly stimulated ROS production) in the presence or absence of hyphae. **(C)** Using heated-killed (HK) hyphae, we demonstrate that live hyphae blocks while HK hyphae stimulates ROS production. Data are expressed as mean ± SEM; *n *= 3, one-way ANOVA with Bonferroni’s posttest. **p < 0.01 ***p < 0.001.

ROS production is a well-described mechanism by which neutrophils and other phagocytes use to kill different types of pathogens. Although ROS production is usually associated with the phagocytosis process, it is known that phagocytes can also release ROS extracellularly, killing unphagocytosed pathogens. Thus, we performed luminol-enhanced chemiluminescence assays to verify whether neutrophils were producing ROS during infections with conidia and hyphae. First, we seeded the neutrophils in the presence of a luminol reagent, and then the cells were then stimulated with medium (negative control), conidia (MOI 1:2), or hyphae (MOI 5:1). After 90 min, the area under the curve was used to calculate the total ROS production. Our data show that conidia stimulated neutrophil ROS production, and hyphae did not (**Figure 2A** and **Supplementary Figure 3A**). In fact, hyphae seem to block ROS production, leading to a level of ROS that was lower than the unstimulated cells. To confirm if hyphae were acting to block ROS production, we stimulated neutrophils with PMA, an agonist of NADPH-oxidase. Neutrophils stimulated with PMA showed a high level of ROS production; however, neutrophils stimulated with PMA in the presence of hyphae showed a statistically lower level of ROS production, confirming that hyphae were acting to block ROS production even in the PMA-stimulated cells (**Figure 2B** and **Supplementary Figure 3B**). Repeating these experiments using heat-killed hyphae, we demonstrated that only live hyphae have the capacity to block ROS production (**Figure 2C** and **Supplementary Figure 3C**).

### ROS Production During Conidial Infection Is TLR-2– and TLR-4–Dependent

**Figure 3:**
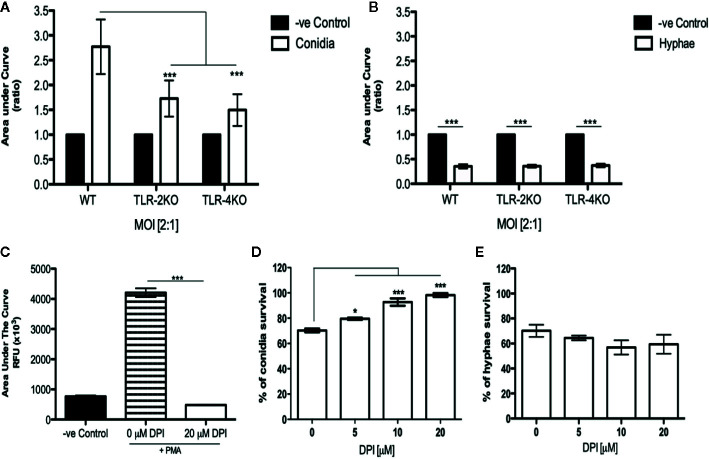
Neutrophil ROS production by *F. pedrosoi* conidia is a mechanism relying on TLR-2 and TLR-4 and essential to conidial killing. WT, TLR-2KO, and TLR-4KO neutrophils were previously purified using Ficoll density layer and then seeded into a 96-well plate in the presence of a luminol reagent and stimulated with *F. pedrosoi* conidia **(A)** or hyphae **(B)**. As unstimulated control, neutrophils were incubated in the absence of fungi to measure the ROS production during the steady state. The ROS production was measured every 2 min to approximately 60 min, and the area under the curve was calculated to measure the total ROS production after stimulation. The area under the curve ratio was calculated using the control of each group (WT, TLR-2KO, or TLR-4KO) and setting as 1. Data are expressed as mean ± SEM; *n *= 5, two-way ANOVA with Bonferroni’s posttest. ***p < 0.001. To verify whether hyphal/conidial killing was dependent on ROS production, we used a range of concentrations of DPI, a potent NADPH-oxidase inhibitor. First, we incubated WT neutrophils with DPI (or not) and stimulated them with PMA. ROS production was measured to confirm the inhibition activity of the drug **(C)**. Then, using a range of DPI concentrations (0–20 μM), we analyze the importance of ROS production in hyphal/conidial killing. After preincubation with DPI, the purified neutrophils were incubated with conidia **(D)** or hyphae **(E)** for 2 h After incubation, the cells were lysed with distilled water, and the supernatant was seeded onto Sabouraud agar at 37°C for 5 days. Data are expressed as mean ± SEM; *n* = 4, one-way ANOVA with Bonferroni’s posttest. *p < 0.05; ***p < 0.001.

Because conidia phagocytosis and killing was impaired in TLR-2KO and TLR-4KO neutrophils, we checked whether the ROS production was affected in these cells. Using the luminol-enhanced chemiluminescence assay, we verified that ROS production was impaired in TLR-2KO and TLR-4KO neutrophils infected with *F. pedrosoi* conidia of (**Figure 3A**). We also verified that the capacity of hyphae to block neutrophil ROS production occurred via a mechanism independent of TLR-2 and TLR-4 (**Figure 3B**).

### ROS Is Essential for Conidia but Not Hyphae Killing 

To confirm that conidia killing was dependent on ROS production, we performed a killing assay using different concentrations of DPI, an NADPH-oxidase inhibitor. First, we performed an ROS assay in the presence (or absence) of 20 μM DPI. Neutrophils were previously incubated with medium (negative control) and 0 μM (DMSO as a vehicle) or DPI (20 μM) for 15 min. Afterward, the cells were stimulated with PMA to confirm that the DPI concentration was able to completely block ROS production (**Figure 3C**). Next, the killing assay was performed using DPI at concentrations ranging from 0 to 20 μM. Our data shows that, in the presence of 20 μM DPI, conidial survival is approximately 95% (**Figure 3D**), and hyphal survival was similar to the control sample (**Figure 3E**). Therefore, we demonstrate that ROS is essential for conidia killing while hyphae killing is a ROS-independent process.

### NET Release During *F. pedrosoi* Hyphae Infection Is TLR-2– and TLR-4–Independent 

**Figure 4:**
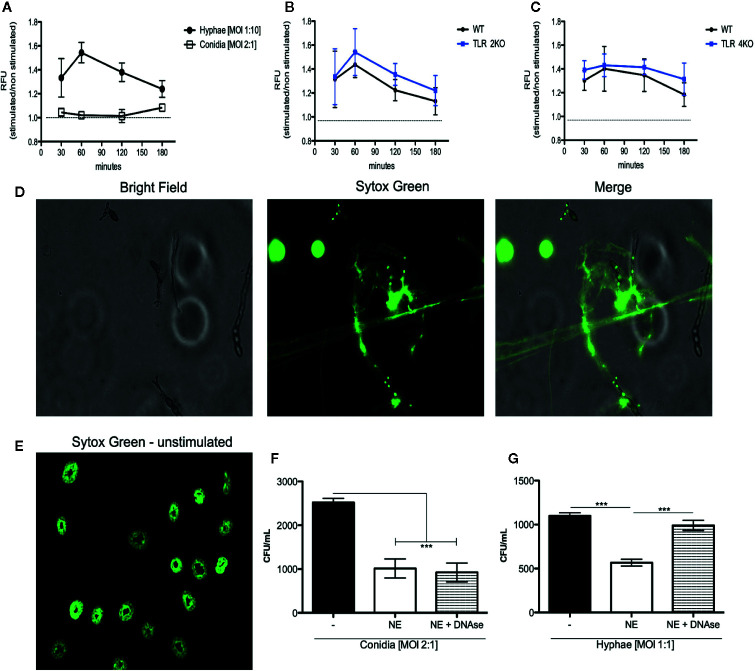
NET release against *F. pedrosoi* hyphae infection is a mechanism independent of TLR-2 and TLR-4 and responsible for hyphae killing. **(A)** WT neutrophils purified by magnetic beads were resuspended in media containing 5 µM styox green dye in resting condition (dashed lines; negative control) or incubated with *F. pedrosoi* hyphae or conidia. Florescence was recorded by a plate reader for every 30 min up to 3 h. DNA release (NETotic index) shows NETosis over hyphae but not conidia infection **(A)**. After 180 min, the neutrophils incubated with hyphae were fixed with 4% (v/v) PFA for 15 min and analyzed by immunofluorescence microscopy (**D** e **E**). Using TLR-2KO **(B)** and TLR-4KO **(C)** neutrophils, we verified that NET release is a mechanism independent from TLR-2KO and TLR-4KO. To confirm that NETs kill *F. pedrosoi* hyphae **(F)** but not conidia **(G)**, WT-purified neutrophils were incubated with *F. pedrosoi* conidia or hyphae in the presence or absence of DNase. After 2 h, the cells were lysed with distilled water and seeded in Sabouraud agar at 37°C for 5 days. Data are expressed as mean ± SEM; *n* = 5, two-way ANOVA with Bonferroni’s posttest. ***p < 0.001.

Although we demonstrate that phagocytosis and ROS production are responsible for conidial killing, these mechanisms are not involved in hyphal killing. Thus, we asked whether hyphae were stimulating NET release. Therefore, a DNA release assay was performed using sytox green to quantify NET release during infection with conidia (MOI 1:2) and hyphae (MOI 5:1). We first verified that hyphae, but not conidia, induce NET release (**Figure 4A**). Then, using TLR-2KO and TLR-4KO neutrophils, we show that these receptors are not responsible for neutrophil activation and NET release (**Figure 4B**, **C**). NET release by WT neutrophils over hyphae was also verified by immunofluorescence microscopy (**Figure 4D** and **Supplementary Figure 4**).

### NET Released by Neutrophils Kills *F. pedrosoi* Hyphae

Although neutrophils are able to release NETs against several pathogens, it is shown that some pathogens are able to degrade or evade killing by NETs. Therefore, to show that the NETs released in response to *F. pedrosoi* hyphae have fungicidal activity and can kill the fungal particles, we performed a killing assay in the presence of DNase. As expected, the survival index of conidia did not change in the presence of DNase (**Figure 4F**), considering that we previously showed that conidia did not stimulate NET release (**Figure 4A**). However, a statistical increase in hyphal survival was shown when NETs were disrupted with DNase, confirming the fungicidal activity of NETs against *F. pedrosoi* hyphae (**Figure 4G**).

### Neutrophil Migration Is Impaired in TLR-2KO and TLR-4KO Animals Infected With *F. pedrosoi*

**Figure 5:**
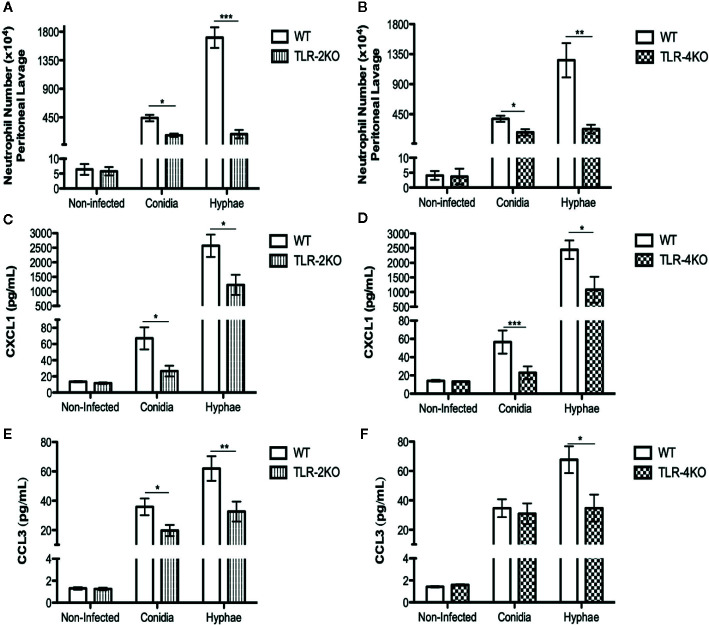
Neutrophil attraction to the conidia- and hyphae-infected site is dependent on TLR-2 and TLR-4 and chemokines CXCL1 and CCL3. WT, TLR-2KO, and TLR-4KO animals were i.p. infected with *F. pedrosoi* (conidia or hyphae) or PBS as noninfected control. After 3 h of infection, animals were euthanized, and the i.p. lavage were collected with PBS 5% FBS 2 mM EDTA. The lavage was centrifuged, and supernatant was collected to measure chemokines by ELISA **(C–F)**. Cells were harvested and counted in a Neubauer chamber for staining with anti-CD45, anti-ly6G, and anti-CD11b to detect neutrophil migration by the flow cytometry technique **(A, B)**. Data are expressed as mean ± SEM; *n* = 9–13, Student *t.* *p < 0.05; **p < 0.01; ***p < 0.001.

Neutrophils are known to be the first cells to migrate to the infection site. To test whether TLR-2 and TLR-4 play a role in neutrophil migration we i.p. infected WT, TLR-2KO, and TLR-4KO animals with conidia and hyphae for 3 h. Afterward, we recovered the migrated cells by i.p. lavage, and cells were counted and stained for flow cytometry analysis. Our results show a higher neutrophil influx during infection with hyphae compared to conidia. Severe impairment in neutrophil migration was observed in animals lacking TLR-2 (**Figure 5A**) and TLR-4 (**Figure 5B**). Because chemokines CXCL1 and CCL3 are known to be important for neutrophil migration, we next measured the levels of CXCL1 (**Figures 5C, D**) and CCL3 (**Figures 5E, F**) in the peritoneal lavage after 3 h of infection. Our data suggest that TLR-2 and TLR-4 are important receptors for sensing *F. pedrosoi*, which stimulate the production of chemokines, such as CXCL1 and CCL3 by peritoneum resident cells. Thus, the impairment of CXCL1 and CCL3 production in TLR-2KO and TLR-4KO animals affects neutrophil migration to the infection site.

### Higher Fungal Burden in Spleen and Liver of TLR-2KO and TLR-4KO Infected Animals

**Figure 6:**
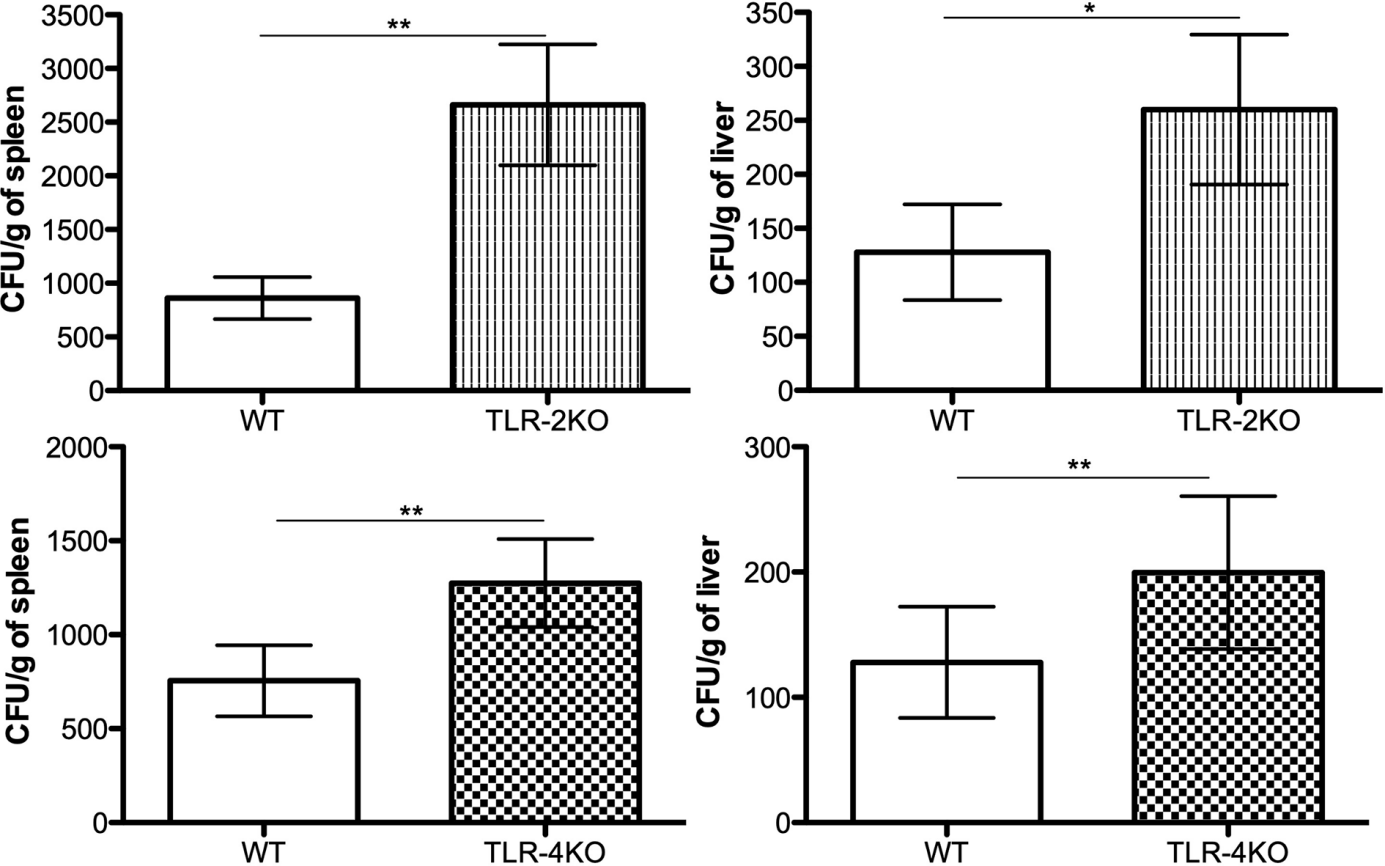
TLR-2 and TLR-4 are essential for fungal load control in early *F. pedrosoi* conidia infection. WT, TLR-2KO, and TLR-4KO animals were infected i.p. with 5x10^7^ *F. pedrosoi* conidia. After 24 h, the animals were euthanized, and the liver and spleen were harvested. An aliquot was seeded onto Sabouraud agar to fungal load analysis. Data are expressed as mean ± SEM; *n* = 10, Student *t.* *p < 0.05; **p < 0.01.

To confirm that TLR-2 and TLR-4 are important in controlling CBM infection in vivo, we i.p. infected WT, TLR-2KO, and TLR-4KO animals with *F. pedrosoi* conidia for 24 h. Afterward, the spleen and liver were harvested, and an aliquot was seeded onto Sabouraud agar plates for later CFU counting. Our results show that animals lacking TLR-2 and TLR-4 had higher fungal loads in the spleen and liver compared to WT animals, confirming that these receptors were also important for controlling the disease in murine models (**Figure 6**). An increase in the neutrophil population was seen in the spleen and liver of the KO animals after infection even though a similar neutrophil population is observed in the noninfected group (**Supplementary Figure 5**).

## Discussion

Currently, CBM treatment has low cure rates and is based on multidrug prescriptions and, in some cases, cryo/heat therapy with surgery (10–14). More effective treatment is needed; therefore, a better understanding of the host–pathogen interaction is crucial. In the past decade, studies showing the importance of C-type lectin receptors (CLR) in fungal infection have increased substantially (35). One of the first studies about CLR response in CBM was published in 2011, in which Sousa Mda G and colleagues demonstrate that *F. pedrosoi* activates the Mincle receptor leading to an anti-inflammatory response, causing a chronic infection. They also demonstrate that the Dectin-1 receptor in lineage macrophages (RAW 264.7) is important to conidia binding; however, they did not observe the same results using mouse primary macrophages. However, they show that Dectin-1 KO mice had a marginal increase in fungal burden, suggesting that Dectin-1 could be important to the control of CBM (36). Three years later, Wevers and colleagues demonstrate that Mincle activation by conidia of *F. pedrosoi* leads to inhibition of Dectin-1 activation by the loss of nuclear IRF1 activity and blockage of IL12A transcription. The absence of IL-12 leads to impaired TH1 responses, promoting TH2 polarization fostering the chronic infection (37). The role of Dectin-2 in *F. pedrosoi* infection was then demonstrated in 2015 by Wuthrich and colleagues (38). They show that Dectin-2 is essential for TH17 polarization, but Mincle activation by *F. pedrosoi* impairs Dectin-2 activation and TH17 polarization. Interestingly, they did not observed an increase in TH1 polarization by Mincle KO mice. De Castro and colleagues later demonstrated that Dectin-1, Dectin-2, and Dectin-3 were important receptors to inflammasome activation, which leads to IL-1β production and hyphae killing (39). Therefore, these studies shed a light on understanding the CLR activation and Mincle suppressive activity over the immune system, leading the infection to a chronic phase. Sousa Mda G also demonstrate that *F. pedrosoi* poorly activate TLRs, showing that exogenous activation of TLRs (i.e., TLR-4 by LPS) would boost the animal immune system helping the control of the disease. Therefore, knowing that TLRs also seem to have an important role in CBM, our study aimed to better understand the role of two of the most important TLRs: TLR-2 and TLR-4. Although several studies demonstrate the essential roles of these receptors (40–42) and neutrophils in several infections (43), including fungal infections (20), the real significance of these receptors and cells in CBM disease has not been fully addressed to date. In the present study, we elucidate the roles of TLR-2 and TLR-4 in several neutrophil effector functions and describe for the first time the mechanism used by neutrophils to kill *F. pedrosoi* hyphae.

The first study of neutrophil function in *F. pedrosoi* infection was carried out in 1996 (34). In that work, the authors demonstrate that conidia were killed by neutrophils through the production of extracellular ROS once a few particles had been detected inside neutrophils. Our study confirms the fungicidal activity of neutrophils toward conidial infection and demonstrates for the first time that neutrophils also have fungicidal activity against *F. pedrosoi* hyphae (**Figure 1A**). Our results also confirm that ROS production is essential for conidia killing (**Figure 3**). However, different from previous studies, we show a high neutrophil phagocytic activity (**Figure 1F** and **Supplementary Figure 2**) and demonstrate that this process is essential for eliminating conidia particles (**Figure 1D**). Although some of our results disagree with previously published data, we have to consider that Rozental and colleagues use rat neutrophils, and some results may be species-specific. Although the vast majority of studies focus on understanding the roles of TLR-2 and TLR-4 in bacterial infections (once these receptors are known to recognize peptidoglycan and lipopolysaccharide, respectively), these receptors are also important in fungal infections because they bind to glucan/mannan and rhamnose, respectively (44, 45). Our findings show that TLR-2 and TLR-4 are essential to conidial but not hyphal killing (**Figure 1B and C**). These receptors were also found to be important for killing *Aspergillus fumigattus* conidia (46) and *Candida albicans* blastoconidia (47); however, *C. albicans* hyphae are recognized by only TLR-2, and *A. fumigatus* hyphae are recognized by only TLR-4 (47). It is known that different forms of the same fungal species may be present in the environment or in the host during an infection process (such as conidia/hyphae or yeast/blastoconidia/pseudohyphae). In addition to their difference in size, the cell-wall components of these different fungal forms can be very different (48), and this seems to be crucial to fungal recognition by the host immune system. Although proteomics studies of the *F. pedrosoi* cell wall have been relatively rare, a couple of studies have demonstrated that conidia and hyphae from *F. pedrosoi* present different cell wall compositions with some similarity in the components but different levels of expression (49, 50). Therefore, although TLR-2 and TLR-4 are crucial in conidial killing, probably due to the difference in cell wall components, these receptors do not play a role in *F pedrosoi* hyphal killing (**Figure 1E**). Using immunofluorescence microscopy, we verified that the absence of TLR-2 and TLR-4 leads to impaired conidial phagocytosis (**Figure 1F** and **Supplementary Figure 2**). Similar results were seen in *P. brasiliensis* (51, 52) and *Sporothrix brasiliensis* (53, 54) infection. In contrast, in *C. albicans* and *A. fumigatus* infection, TLR-4 does not play a crucial role in the phagocytic process (55, 56).

Although phagocytosis is an important neutrophil effector function, it is not sufficient for particle killing. For that, the phagosome has to fuse with the lysosome so that the oxidative burst can take place. This leads to ROS production, which is responsible for the killing of phagocytosed pathogens. However, it is well described that phagocytes can also release extracellular ROS (phagocytosis-independently), making this a possible mechanism of extracellular hyphal and conidial killing (57). Based on that, we asked whether conidia and hyphae were stimulating neutrophil ROS production. Our findings show that neutrophils produce ROS during conidial infection in a TLR-2– and TLR-4–dependent manner (**Figure 3A**). In contrast to our findings, TLR-2 and TLR-4 are not involved in phagocytosis (56) and ROS production in *C. albicans* infection (58) although these receptors are essential to control the infection in vivo. Although our results demonstrate that neutrophils produce ROS in a conidial infection, we cannot ensure whether ROS production is dependent on phagocytosis or not. Because our data demonstrate that TLR-2 and TLR-4 are important for conidial phagocytosis, we believe that the impairment in ROS production in TLR-2KO and TLR-4KO are related to its lower phagocytosis index (**Figure 1F** and **Supplementary Figure 2**). Therefore, we cannot rule out that TLR-2 and TLR-4 might be important for conidial recognition and extracellular-ROS release in a phagocytosis-independent manner.

We also demonstrate that *F. pedrosoi* hyphae do not stimulate neutrophil ROS production (**Figure 2A**). In fact, we observed that neutrophils infected with hyphae were producing lower amounts of ROS than resting neutrophils (unstimulated). Some studies demonstrate that conidia have the capacity to block nitric oxide (NO) production even in IFN-γ-stimulated murine macrophages (59, 60). Even though conidia and melanin purified from conidia were shown to block NO production, these particles were found to stimulate macrophage ROS production. However, the authors did not show the basal levels of ROS production in healthy animals (uninfected); therefore, we cannot conclude whether the hyphae were weakly stimulating ROS or even not stimulating or blocking ROS production (61). Thus, by preactivating bone marrow–purified neutrophils with PMA, we show that hyphae block neutrophil ROS production (**Figure 2B**). This inhibition is lost when the hyphae are heat killed (**Figure 2C**). Similar results were found with *Aspergillus nidulans* hyphae, and no ROS was produced by infected neutrophils. The authors verified that *Aspergillus nidulans* hyphae killing by NADPH oxidase–deficient neutrophils (from patients with granulomatous chronic disease) was similar to healthy neutrophils, demonstrating that neutrophils kill *A. nidulans* hyphae in an ROS-independent manner (62). Our findings also show that TLR-2 and TLR-4 are not involved in the blocking of ROS production by *F. pedrosoi* hyphae (**Figure 3B**) and that hyphae are killed by an ROS-independent mechanism (**Figure 3E**). Even though the first description of NET release suggests that this activity was dependent on ROS production by NADPH oxidase (63), several recent studies demonstrate that NET release can be an NADPH oxidase–dependent or –independent process (64–66). An important study demonstrates that neutrophils can sense the size of a pathogen to decide whether the cells will phagocytose or release NETs to kill the pathogen (67). However, pathogen size is not the only feature that leads to neutrophil activation and NET release because some bacteria (63) and yeast (22) stimulate neutrophil NET release even though they are small enough to be phagocytosed. Based on that, we asked whether *F. pedrosoi* conidia and hyphae were stimulating NET release. Our findings show that neutrophils release NETs in response to *F. pedrosoi* hyphae but not conidia. Unlikely phagocytosis, NET release occurs via a TLR-2 and TLR-4 independent mechanism (**Figure 4**). Our data suggest that NET release over *F. pedrosoi* hyphae is a NOX-independent process. We next performed a single experiment of NET release in the presence of DPI and observed a strong impairment in PMA-stimulated NET release in the presence of DPI. However, hyphae-stimulated NETs release is not affected by DPI (**Supplementary Figure 6**). Taken together, these results suggest that *F. pedrosoi* hyphae stimulate NETs release in a NOX-independent pathway. Although several studies have been published showing the NET release over several fungi infections, only a few focus on verifying which TLRs or CLRs were involved in the NET release. The receptors related to NET release seem to be pathogen specific. It is demonstrated that TLR-2 and TLR-4 are essential to NET release over *C. albicans* but not over *P. brasiliensis* and *A. fumigatus*. Although Dectin-1 is shown to be important in NET release over *C. albicans* and *P. brasiliensis* but not over *A. fumigatus* (68–70). Therefore, more studies need to be done to understand which receptors are stimulating NET release by murine neutrophils over hyphae structure of *F. pedrosoi*. We believe that Dectin family receptors are one of the most probable candidates involved in this neutrophil effector function. Although neutrophils release NET fibers during several pathogen infections, different studies demonstrate that some pathogens can evade NET killing by degrading the fibers through the formation of biofilms or as a result of the presence of the extracellular capsule (71). Therefore, we performed a killing assay with DNase and demonstrated for the first time that NETs are a mechanism used by neutrophils to eliminate *F. pedrosoi* hyphae (**Figure 4G**). Our data demonstrate that neutrophils act to distinguish conidia or hyphae infection, and we believe that this neutrophil fate could be interfering with granuloma formation in CBM patients. Siqueira and colleagues demonstrate that hyphae infection would lead to granuloma formation in mice CMB, but this granuloma was rarely seen in conidia infection (72). We believe that the differences in neutrophil fate could be interfering with the skin lesions and with granuloma formation. Clearance of apoptotic neutrophil by macrophages is a “silent” process, which does not cause tissue injury or activation of other immune cells. However, neutrophils undergone to an uncontrolled NETosis process would lead to tissue damage and wound-healing defectiveness. NET clearance by macrophages was also shown to be a pro-inflammatory process that leads to tissue damage. Therefore, the uncontrolled stimulation of NETs by hyphae of *F. pedrosoi* might be playing an important role in patients’ lesions as ulceration and increase of the fibrosis process. Therefore, we believe that, after killing conidia and hyphae of *F. pedrosoi*, the distinctive type of neutrophil death will lead to a specific clearance by macrophages that will affect the lesion microenvironmental and skin tissue.

In vivo experiments were also performed to evaluate the roles of TLR-2 and TLR-4 in a murine CBM infection model. First, we verified an impairment in neutrophil migration to the infection site in animals lacking TLR-2 and TLR-4 in both conidial and hyphal infections (**Figure 5A**, **B**). Then, we showed a lower level of CXCL1 and CCL3 in TLR-2KO and TLR-4KO peritoneal lavage, suggesting that TLR-2 and TLR-4 are important to chemokine production by resident cells in the infection site. Therefore, our data demonstrate that TLR-2 and TLR-4 are indirectly associated with neutrophil migration because the lower levels of CXCL1 and CCL3 in KO animals’ infection site led to an impairment in neutrophil migration (**Figures 5C–F**). Similar results were observed in *A. fumigatus* infection, where TLR-2KO and TLR-4KO macrophages released lower levels of the MIP-2 chemokine, resulting in a decrease in neutrophil migration (73). In *C. albicans* infection, animals lacking TLR-4 showed lower levels of MIP-2 and KC leading to impaired neutrophil migration to the infection site (56). At least in our hands, conidia and hyphae seem not to stimulate a direct neutrophil migration in vitro (transwell assays; data not shown). Therefore, the importance of neutrophil’s TLR-2 and TLR-4 to its migration is yet to be determined. Finally, our study shows that TLR-2 and TLR-4 are important in controlling acute CBM infection because animals lacking these receptors had higher spleen and liver fungal loads (**Figure 6**).

In summary, our results show for the first time that neutrophils are important for *F. pedrosoi* conidia and hyphae killing. The cell wall composition and pathogen size may be acting to modulate neutrophil function, leading to phagocytosis and ROS production during conidial infection while ROS-independent NET release is the main effector function involved in hyphal killing. We also demonstrate that TLR-2 and TLR-4 are important receptors in recognition of conidia but not in recognition or killing of hyphae. These receptors were also crucial for neutrophil migration toward the infection site and in the control of the fungal burden in the animals. Therefore, our findings help to better understand the physiopathology of CBM and how the neutrophils fight against *F. pedrosoi* conidia and hyphae infection.

## Data Availability Statement

All datasets generated for this study are included in the article/**Supplementary Material**.

## Ethics Statement

The animal study was reviewed and approved by Comissão de Ética no Uso de Animais da Faculdade de Ciências Farmacêuticas da Universidade de São Paulo

## Author Contributions

LB and SA designed the research, interpreted the data, and wrote the manuscript. LB, CB, JA, LP, GJ, IM, and RA performed the experiments. NC and KF provided scientific input. All authors contributed to the article and approved the submitted version.

## Funding

This work was supported by the Fundação de Amparo à Pesquisa do Estado de São Paulo (FAPESP - process 2012/18598-7, 2014/11146-9, 2016/047293), Coordenação de Aperfeiçoamento de Pessoal de Nível Superior (CAPES), and Conselho Nacional de Desenvolvimento Científico e Tecnológico (CNPq).

This manuscript has been released as a preprint at www.biorxiv.org (Breda et al. 2020) (74).

## Conflict of Interest

The authors declare that the research was conducted in the absence of any commercial or financial relationships that could be construed as a potential conflict of interest.

## Supplementary Material

Supplementary Figure 1 Bone marrow neutrophil purity. Bone marrow cells were harvested, and neutrophils were enriched by Ficoll density **(A)** or using the microbeads anti-Ly-6G ultraPure positive selection kit (Miltenyi Biotec^®^) **(B)**. After enrichment, cells were stained with anti-Ly6G (APC) antibodies and analyzed by flow cytometry. Neutrophil purity ranged from 85% to 98%.

Supplementary Figure 2 Conidia phagocytosis is a TLR-2– and TLR-4–dependent process. WT, TLR-2KO, and TLR-4KO neutrophils were previously purified by Ficoll density layer and then incubated with *F. pedrosoi* conidia (MOI 1:4) for 120 min. After incubation, the cells were washed and fixed with 4% PFA for 15 min followed by permeabilized with 0.01% PBS-T for 10 min. After washing, the neutrophil nuclei were stained with sytox green, and the slides were mounted using Vecta-Shield^®^ and sealed with nail polish. At least 100 cells were analyzed to calculate the phagocytosis index shown in **Figure 1**.

Supplementary Figure 3 Time course of neutrophil ROS production is stimulated by *F. pedrosoi* conidia and blocked by *F. pedrosoi* hyphae. **(A)** WT neutrophils purified by Ficoll were seeded in a 96-well plate in the presence of a luminol reagent and stimulated with *F. pedrosoi* conidia or hyphae. The ROS production was measured every 2 min to approximately 60 min. As unstimulated control, neutrophils were incubated in the absence of fungi to measure the ROS production during the steady state. The area under the curve was calculated to measure the total ROS production after 60 min. **(B)** To confirm that hyphae block ROS production, we stimulated the cells with PMA (highly stimulated ROS production) in the presence or absence of hyphae. **(C)** Using heated-killed (HK) hyphae, we demonstrated that live hyphae blocks, and HK hyphae stimulates ROS production. Data are expressed as mean ± SEM; *n* = 3. Area under the curve was calculated to quantify the total amount of ROS production.

Supplementary Figure 4 Immunofluorescence of NETs stimulated by hyphae of *F. pedrosoi*. WT neutrophils were resuspended in media (control) or incubated with *F. pedrosoi* hyphae for 180 min. Afterward, neutrophils were fixed with 4% (v/v) PFA for 15 min, followed by permeabilization with PBS-T for 15 min. Cells were then incubated with antihistone 3 antibody for 1 h followed by incubation with secondary antibody conjugated with alexa-fluor 647 and sytox green dye. After washing, slides were mounted in 5 μL Vecta-Shield^®^ and sealed with nail polish.

Supplementary Figure 5 Increase of neutrophil population in spleen and liver of TLR-2KO and TLR-4KO animals. Animals were infected i.p. with 5x10^7^ conidia of *F. pedrosoi* or PBS (noninfected group). After 24 h, the animals were euthanized, and the spleen **(A** and **B)** and liver **(C** and **D)** were collected for neutrophil analysis by flow cytometry. Peripheral blood from the noninfected group was also collected to verify neutrophil profile in the steady-state condition of WT, TLR-2KO, and TLR-4KO animals **(E** and **F)**. Data are expressed as mean ± SEM; *n* = 3–10, two-way ANOVA with Bonferroni’s posttest. **p* < 0.05; ***p* < 0.01; ****p* < 0.001.

Supplementary Figure 6 NET release in *F. pedrosoi* hyphae infection is a mechanism independent of NADPH oxidase. WT neutrophils were resuspended in media containing 5 µM sytox green dye in resting condition (dashed lines; negative control) or incubated with PMA or *F. pedrosoi* hyphae in the presence or absence of 20 μM DPI. After 180 mins, fluorescence was measured and the NETotic index was calculated. In the presence of DPI (20 μM) NET release was deeply inhibited in PMA but not in hyphae-stimulated neutrophils. Data are expressed as mean ± SEM; *n*=2.

## References

[1] Queiróz AJR Pereira Domingos F Antônio JR Chromoblastomycosis: clinical experience and review of literature. Int J Dermatol (2018) 57(11):1351–5. 10.1111/ijd.14185 30113072

[2] Caligiorne RB de Resende MA Dias-Neto E Oliveira SC Azevedo V Dematiaceous fungal pathogens: analysis of ribosomal DNA gene polymorphism by polymerase chain reaction-restriction fragment length polymorphism. Mycoses (1999) 42(11-12):609–14. 10.1046/j.1439-0507.1999.00527.x 10680436

[3] Brandt ME Warnock DW Epidemiology, clinical manifestations, and therapy of infections caused by dematiaceous fungi. J Chemother (2003) 15 Suppl 2:36–47. 10.1179/joc.2003.15.Supplement-2.36 14708965

[4] Silva JP de Souza W Rozental S Chromoblastomycosis: a retrospective study of 325 cases on Amazonic Region (Brazil). Mycopathologia (1998) 143(3):171–5. 10.1023/A:1006957415346 10353215

[5] Minotto R Bernardi CD Mallmann LF Edelweiss MI Scroferneker ML Chromoblastomycosis: a review of 100 cases in the state of Rio Grande do Sul, Brazil. J Am Acad Dermatol (2001) 44(4):585–92. 10.1067/mjd.2001.112220 11260530

[6] Bonifaz A Carrasco-Gerard E Saúl A Chromoblastomycosis: clinical and mycologic experience of 51 cases. Mycoses (2001) 44(1-2):1–7. 10.1046/j.1439-0507.2001.00613.x 11398635

[7] Queiroz-Telles F de Hoog S Santos DW Salgado CG Vicente VA Bonifaz A Chromoblastomycosis. Clin Microbiol Rev (2017) 30(1):233–76. 10.1128/CMR.00032-16 PMC5217794 27856522

[8] Esterre P Andriantsimahavandy A Ramarcel ER Pecarrere JL Forty years of chromoblastomycosis in Madagascar: a review. Am J Trop Med Hyg (1996) 55(1):45–7. 10.4269/ajtmh.1996.55.45 8702021

[9] Esterre P Queiroz-Telles F Management of chromoblastomycosis: novel perspectives. Curr Opin Infect Dis (2006) 19(2):148–52. 10.1097/01.qco.0000216625.28692.67 16514339

[10] Bonifaz A Paredes-Solís V Saúl A Treating chromoblastomycosis with systemic antifungals. Expert Opin Pharmacother (2004) 5(2):247–54. 10.1517/14656566.5.2.247 14996622

[11] Castro LG Pimentel ER Lacaz CS Treatment of chromomycosis by cryosurgery with liquid nitrogen: 15 years’ experience. Int J Dermatol (2003) 42(5):408–12. 10.1046/j.1365-4362.2003.01532.x 12755986

[12] Lupi O Tyring SK McGinnis MR Tropical dermatology: fungal tropical diseases. J Am Acad Dermatol (2005) 53(6):931–51, quiz 52-4. 10.1016/j.jaad.2004.10.883 16310053

[13] Krzyściak PM Pindycka-Piaszczyńska M Piaszczyński M Chromoblastomycosis. Postepy Dermatol Alergol (2014) 31(5):310–21. 10.5114/pdia.2014.40949 PMC4221348 25395928

[14] Brito AC Bittencourt MJS Chromoblastomycosis: an etiological, epidemiological, clinical, diagnostic, and treatment update. Bras Dermatol (2018) 93(4):495–506. 10.1590/abd1806-4841.20187321 PMC6063100 30066754

[15] Mazo Fávero Gimenes V Da Glória de Souza M Ferreira KS Marques SG Gonçalves AG Vagner de Castro Lima Santos D Cytokines and lymphocyte proliferation in patients with different clinical forms of chromoblastomycosis. Microbes Infect (2005) 7(4):708–13. 10.1016/j.micinf.2005.01.006 15848277

[16] Sousa MG de Maria Pedrozo e Silva Azevedo C Nascimento RC Ghosn EE Santiago KL Noal V Fonsecaea pedrosoi infection induces differential modulation of costimulatory molecules and cytokines in monocytes from patients with severe and mild forms of chromoblastomycosis. J Leukoc Biol (2008) 84(3):864–70. 10.1189/jlb.0308211 18562487

[17] Teixeira de Sousa Ma G Ghosn EE Almeida SR Absence of CD4+ T cells impairs host defence of mice infected with Fonsecaea pedrosoi. Scand J Immunol (2006) 64(6):595–600. 10.1111/j.1365-3083.2006.01846.x 17083615

[18] Farbiarz SR De Carvalho TU Alviano C De Souza W Fine structure and cytochemistry of the interaction between Fonsecaea pedrosoi and mouse resident macrophages. J Med Vet Mycol (1990) 28(5):373–83. 10.1080/02681219080000481 2283584

[19] Rozental S Alviano CS de Souza W The in vitro susceptibility of Fonsecaea pedrosoi to activated macrophages. Mycopathologia (1994) 126(2):85–91. 10.1007/BF01146200 8065435

[20] Rosales C Neutrophil: A Cell with Many Roles in Inflammation or Several Cell Types? Front Physiol (2018) 9:113. 10.3389/fphys.2018.00113 29515456 PMC5826082

[21] Eskan MA Jotwani R Abe T Chmelar J Lim JH Liang S The leukocyte integrin antagonist Del-1 inhibits IL-17-mediated inflammatory bone loss. Nat Immunol (2012) 13(5):465–73. 10.1038/ni.2260 PMC3330141 22447028

[22] Weaver CT Elson CO Fouser LA Kolls JK The Th17 pathway and inflammatory diseases of the intestines, lungs, and skin. Annu Rev Pathol (2013) 8:477–512. 10.1146/annurev-pathol-011110-130318 23157335 PMC3965671

[23] Chen F Wu W Millman A Craft JF Chen E Patel N Neutrophils prime a long-lived effector macrophage phenotype that mediates accelerated helminth expulsion. Nat Immunol (2014) 15(10):938–46. 10.1038/ni.2984 PMC4479254 25173346

[24] Kruger P Saffarzadeh M Weber AN Rieber N Radsak M von Bernuth H Neutrophils: Between host defence, immune modulation, and tissue injury. PloS Pathog (2015) 11(3):e1004651. 10.1371/journal.ppat.1004651 25764063 PMC4357453

[25] Segel GB Halterman MW Lichtman MA The paradox of the neutrophil’s role in tissue injury. J Leukoc Biol (2011) 89(3):359–72. 10.1189/jlb.0910538 PMC6608002 21097697

[26] de Souza CN Breda LCD Khan MA de Almeida SR Saraiva Camara NO Sweezey N Alkaline pH Promotes NADPH Oxidase-Independent Neutrophil Extracellular Trap Formation: A Matter of Mitochondrial Reactive Oxygen Species Generation and Citrullination and Cleavage of Histone. Front Immunol (2018) 8. 10.3389/fimmu.2017.01849 PMC5767187 29375550

[27] Gazendam RP van de Geer A Roos D van den Berg TK Kuijpers TW How neutrophils kill fungi. Immunol Rev (2016) 273(1):299–311. 10.1111/imr.12454 27558342

[28] Gazendam RP van Hamme JL Tool AT Hoogenboezem M van den Berg JM Prins JM Human Neutrophils Use Different Mechanisms To Kill Aspergillus fumigatus Conidia and Hyphae: Evidence from Phagocyte Defects. J Immunol (2016) 196(3):1272–83. 10.4049/jimmunol.1501811 26718340

[29] Rocha JD Nascimento MT Decote-Ricardo D Côrte-Real S Morrot A Heise N Capsular polysaccharides from Cryptococcus neoformans modulate production of neutrophil extracellular traps (NETs) by human neutrophils. Sci Rep (2015) 5:8008. 10.1038/srep08008 25620354 PMC4306120

[30] Rodrigues DR Dias-Melicio LA Calvi SA Peraçoli MT Soares AM Paracoccidioides brasiliensis killing by IFN-gamma, TNF-alpha and GM-CSF activated human neutrophils: role for oxygen metabolites. Med Mycol (2007) 45(1):27–33. 10.1080/13693780600981676 17325941

[31] Rex JH Bennett JE Administration of potassium iodide to normal volunteers does not increase killing of Sporothrix schenckii by their neutrophils or monocytes. J Med Vet Mycol (1990) 28(3):185–9. 10.1080/02681219080000241 2120415

[32] Santos AL Palmeira VF Rozental S Kneipp LF Nimrichter L Alviano DS Biology and pathogenesis of Fonsecaea pedrosoi, the major etiologic agent of chromoblastomycosis. FEMS Microbiol Rev (2007) 31(5):570–91. 10.1111/j.1574-6976.2007.00077.x 17645522

[33] Correia RT Valente NY Criado PR Martins JE Chromoblastomycosis: study of 27 cases and review of medical literature. Bras Dermatol (2010) 85(4):448–54. 10.1590/S0365-05962010000400005 20944904

[34] Rozental S Alviano CS de Souza W Fine structure and cytochemical study of the interaction between Fonsecaea pedrosoi and rat polymorphonuclear leukocyte. J Med Vet Mycol (1996) 34(5):323–30. 10.1080/02681219680000551 8912165

[35] Hardison SE Brown GD C-type lectin receptors orchestrate antifungal immunity. Nat Immunol (2012) 13(9):817–22. 10.1038/ni.2369 PMC3432564 22910394

[36] Sousa MAG Reid DM Schweighoffer E Tybulewicz V Ruland J Langhorne J Restoration of pattern recognition receptor costimulation to treat chromoblastomycosis, a chronic fungal infection of the skin. Cell Host Microbe (2011) 9(5):436–43. 10.1016/j.chom.2011.04.005 PMC3098964 21575914

[37] Wevers BA Kaptein TM Zijlstra-Willems EM Theelen B Boekhout T Geijtenbeek TB Fungal engagement of the C-type lectin mincle suppresses dectin-1-induced antifungal immunity. Cell Host Microbe (2014) 15(4):494–505. 10.1016/j.chom.2014.03.008 24721577

[38] Wüthrich M Wang H Li M Lerksuthirat T Hardison SE Brown GD Fonsecaea pedrosoi-induced Th17-cell differentiation in mice is fostered by Dectin-2 and suppressed by Mincle recognition. Eur J Immunol (2015) 45(9):2542–52. 10.1002/eji.201545591 PMC4562893 26140582

[39] de Castro RJA Siqueira IM Jerônimo MS Basso AMM Veloso Junior PHH Magalhães KG The Major Chromoblastomycosis Etiologic Agent. Front Immunol (2017) 8:572. 10.3389/fimmu.2017.01572 29209318 PMC5702042

[40] Kawai T Akira S Toll-like receptors and their crosstalk with other innate receptors in infection and immunity. Immunity (2011) 34(5):637–50. 10.1016/j.immuni.2011.05.006 21616434

[41] Kumar H Kawai T Akira S Toll-like receptors and innate immunity. Biochem Biophys Res Commun (2009) 388(4):621–5. 10.1016/j.bbrc.2009.08.062 19686699

[42] Mukherjee S Karmakar S Babu SP TLR2 and TLR4 mediated host immune responses in major infectious diseases: a review. Braz J Infect Dis (2016) 20(2):193–204. 10.1016/j.bjid.2015.10.011 26775799 PMC9427569

[43] Segal AW How neutrophils kill microbes. Annu Rev Immunol (2005) 23:197–223. 10.1146/annurev.immunol.23.021704.115653 15771570 PMC2092448

[44] Akira S Uematsu S Takeuchi O Pathogen recognition and innate immunity. Cell (2006) 124(4):783–801. 10.1016/j.cell.2006.02.015 16497588

[45] Oliva C Turnbough CL Kearney JF CD14-Mac-1 interactions in Bacillus anthracis spore internalization by macrophages. Proc Natl Acad Sci U S A (2009) 106(33):13957–62. 10.1073/pnas.0902392106 PMC2729002 19666536

[46] Netea MG Warris A Van der Meer JW Fenton MJ Verver-Janssen TJ Jacobs LE Aspergillus fumigatus evades immune recognition during germination through loss of toll-like receptor-4-mediated signal transduction. J Infect Dis (2003) 188(2):320–6. 10.1086/376456 12854089

[47] van der Graaf CA Netea MG Verschueren I van der Meer JW Kullberg BJ Differential cytokine production and Toll-like receptor signaling pathways by Candida albicans blastoconidia and hyphae. Infect Immun (2005) 73(11):7458–64. 10.1128/IAI.73.11.7458-7464.2005 PMC1273874 16239547

[48] Erwig LP Gow NA Interactions of fungal pathogens with phagocytes. Nat Rev Microbiol (2016) 14(3):163–76. 10.1038/nrmicro.2015.21 26853116

[49] Gomes MH Resende MA Fonsecaea pedrosoi: lipid composition and determination of susceptibility to amphotericin B. Can J Microbiol (1992) 38(3):209–14. 10.1139/m92-035 1393822

[50] de A Soares RM Angluster J de Souza W Alviano CS Carbohydrate and lipid components of hyphae and conidia of human pathogen Fonsecaea pedrosoi. Mycopathologia (1995) 132(2):71–7. 10.1007/BF01103778 8819829

[51] Acorci-Valério MJ Bordon-Graciani AP Dias-Melicio LA de Assis Golim M Nakaira-Takahagi E de Campos Soares AM Role of TLR2 and TLR4 in human neutrophil functions against Paracoccidioides brasiliensis. Scand J Immunol (2010) 71(2):99–108. 10.1111/j.1365-3083.2009.02351.x 20384861

[52] Calich VL Pina A Felonato M Bernardino S Costa TA Loures FV Toll-like receptors and fungal infections: the role of TLR2, TLR4 and MyD88 in paracoccidioidomycosis. FEMS Immunol Med Microbiol (2008) 53(1):1–7. 10.1111/j.1574-695X.2008.00378.x 18384366

[53] Rossato L Silvana Dos Santos S Ferreira LG Rogério de Almeida S The impact of the absence of Toll-like receptor-2 during Sporothrix brasiliensis infection. J Med Microbiol (2019) 68(1):87–94. 10.1099/jmm.0.000876 30451650

[54] Rossato L Santos SSD Ferreira LG de Almeida SR The importance of Toll-like receptor 4 during experimental Sporothrix brasiliensis infection. Med Mycol (2019) 57(4):489–95. 10.1093/mmy/myy048 30085101

[55] Chai LY Kullberg BJ Vonk AG Warris A Cambi A Latgé JP Modulation of Toll-like receptor 2 (TLR2) and TLR4 responses by Aspergillus fumigatus. Infect Immun (2009) 77(5):2184–92. 10.1128/IAI.01455-08 PMC2681752 19204090

[56] Netea MG Van Der Graaf CA Vonk AG Verschueren I Van Der Meer JW Kullberg BJ The role of toll-like receptor (TLR) 2 and TLR4 in the host defense against disseminated candidiasis. J Infect Dis (2002) 185(10):1483–9. 10.1086/340511 11992285

[57] Bylund J Brown KL Movitz C Dahlgren C Karlsson A Intracellular generation of superoxide by the phagocyte NADPH oxidase: how, where, and what for? Free Radic Biol Med (2010) 49(12):1834–45. 10.1016/j.freeradbiomed.2010.09.016 20870019

[58] Villamón E Gozalbo D Roig P O’Connor JE Fradelizi D Gil ML Toll-like receptor-2 is essential in murine defenses against Candida albicans infections. Microbes Infect (2004) 6(1):1–7. 10.1016/j.micinf.2003.09.020 14738887

[59] Hayakawa M Ghosn EE da Gloria Teixeria de Sousa M Ferreira KS Almeida SR Phagocytosis, production of nitric oxide and pro-inflammatory cytokines by macrophages in the presence of dematiaceous [correction of dematiaceus] fungi that cause chromoblastomycosis. Scand J Immunol (2006) 64(4):382–7. 10.1111/j.1365-3083.2006.01804.x 16970678

[60] Bocca AL Brito PP Figueiredo F Tosta CE Inhibition of nitric oxide production by macrophages in chromoblastomycosis: a role for Fonsecaea pedrosoi melanin. Mycopathologia (2006) 161(4):195–203. 10.1007/s11046-005-0228-6 16552481

[61] Dong B Tong Z Li R Chen SC Liu W Chen Y Transformation of Fonsecaea pedrosoi into sclerotic cells links to the refractoriness of experimental chromoblastomycosis in BALB/c mice via a mechanism involving a chitin-induced impairment of IFN-γ production. PloS Negl Trop Dis (2018) 12(2):e0006237. 10.1371/journal.pntd.0006237 29481557 PMC5843349

[62] Henriet SS Hermans PW Verweij PE Simonetti E Holland SM Sugui JA Human leukocytes kill Aspergillus nidulans by reactive oxygen species-independent mechanisms. Infect Immun (2011) 79(2):767–73. 10.1128/IAI.00921-10 PMC3028853 21078850

[63] Brinkmann V Reichard U Goosmann C Fauler B Uhlemann Y Weiss DS Neutrophil extracellular traps kill bacteria. Science (2004) 303(5663):1532–5. 10.1126/science.1092385 15001782

[64] Douda DN Khan MA Grasemann H Palaniyar N SK3 channel and mitochondrial ROS mediate NADPH oxidase-independent NETosis induced by calcium influx. Proc Natl Acad Sci U S A (2015) 112(9):2817–22. 10.1073/pnas.1414055112 PMC4352781 25730848

[65] Fuchs TA Abed U Goosmann C Hurwitz R Schulze I Wahn V Novel cell death program leads to neutrophil extracellular traps. J Cell Biol (2007) 176(2):231–41. 10.1083/jcb.200606027 PMC2063942 17210947

[66] Yipp BG Kubes P NETosis: how vital is it? Blood (2013) 122(16):2784–94. 10.1182/blood-2013-04-457671 24009232

[67] Branzk N Lubojemska A Hardison SE Wang Q Gutierrez MG Brown GD Neutrophils sense microbe size and selectively release neutrophil extracellular traps in response to large pathogens. Nat Immunol (2014) 15(11):1017–25. 10.1038/ni.2987 PMC4236687 25217981

[68] Zawrotniak M Bochenska O Karkowska-Kuleta J Seweryn-Ozog K Aoki W Ueda M Aspartic Proteases and Major Cell Wall Components in. Front Cell Infect Microbiol (2017) 7:414. 10.3389/fcimb.2017.00414 28983472 PMC5613151

[69] Bachiega TF Dias-Melicio LA Fernandes RK de Almeida Balderramas H Rodrigues DR Ximenes VF Participation of dectin-1 receptor on NETs release against Paracoccidioides brasiliensis: Role on extracellular killing. Immunobiology (2016) 221(2):228–35. 10.1016/j.imbio.2015.09.003 26416210

[70] Silva JC Rodrigues NC Thompson-Souza GA Muniz VS Neves JS Figueiredo RT Mac-1 triggers neutrophil DNA extracellular trap formation to Aspergillus fumigatus independently of PAD4 histone citrullination. J Leukoc Biol (2020) 107(1):69–83. 10.1002/JLB.4A0119-009RR 31478251

[71] Storisteanu DM Pocock JM Cowburn AS Juss JK Nadesalingam A Nizet V Evasion of Neutrophil Extracellular Traps by Respiratory Pathogens. Am J Respir Cell Mol Biol (2017) 56(4):423–31. 10.1165/rcmb.2016-0193PS PMC5449512 27854516

[72] Siqueira IM de Castro RJA Leonhardt LCM Jerônimo MS Soares AC Raiol T Modulation of the immune response by Fonsecaea pedrosoi morphotypes in the course of experimental chromoblastomycosis and their role on inflammatory response chronicity. PloS Negl Trop Dis (2017) 11(3):e0005461. 10.1371/journal.pntd.0005461 28355277 PMC5391973

[73] Meier A Kirschning CJ Nikolaus T Wagner H Heesemann J Ebel F Toll-like receptor (TLR) 2 and TLR4 are essential for Aspergillus-induced activation of murine macrophages. Cell Microbiol (2003) 5(8):561–70. 10.1046/j.1462-5822.2003.00301.x 12864815

[74] Breda LCD de Souza Breda CN de Almeida JRF Paulo LNM Jannuzzi GP de Godoy Menezes I Fonsecaea pedrosoi conidia and hyphae activate neutrophils distinctly: Requirement of TLR-2 and TLR-4 in neutrophil effector functions. bioRxiv (2020). 10.1101/2020.01.06.895706 PMC7609859 33193308

