# Supplementary figures and images for: Fonsecaea pedrosoi Conidia and Hyphae Activate Neutrophils Distinctly: Requirement of TLR-2 and TLR-4 in Neutrophil Effector Functions
Source: Front Immunol. 2020 Oct 21;11:540064. doi: 10.3389/fimmu.2020.540064 (PMC7609859; doi:10.3389/fimmu.2020.540064)

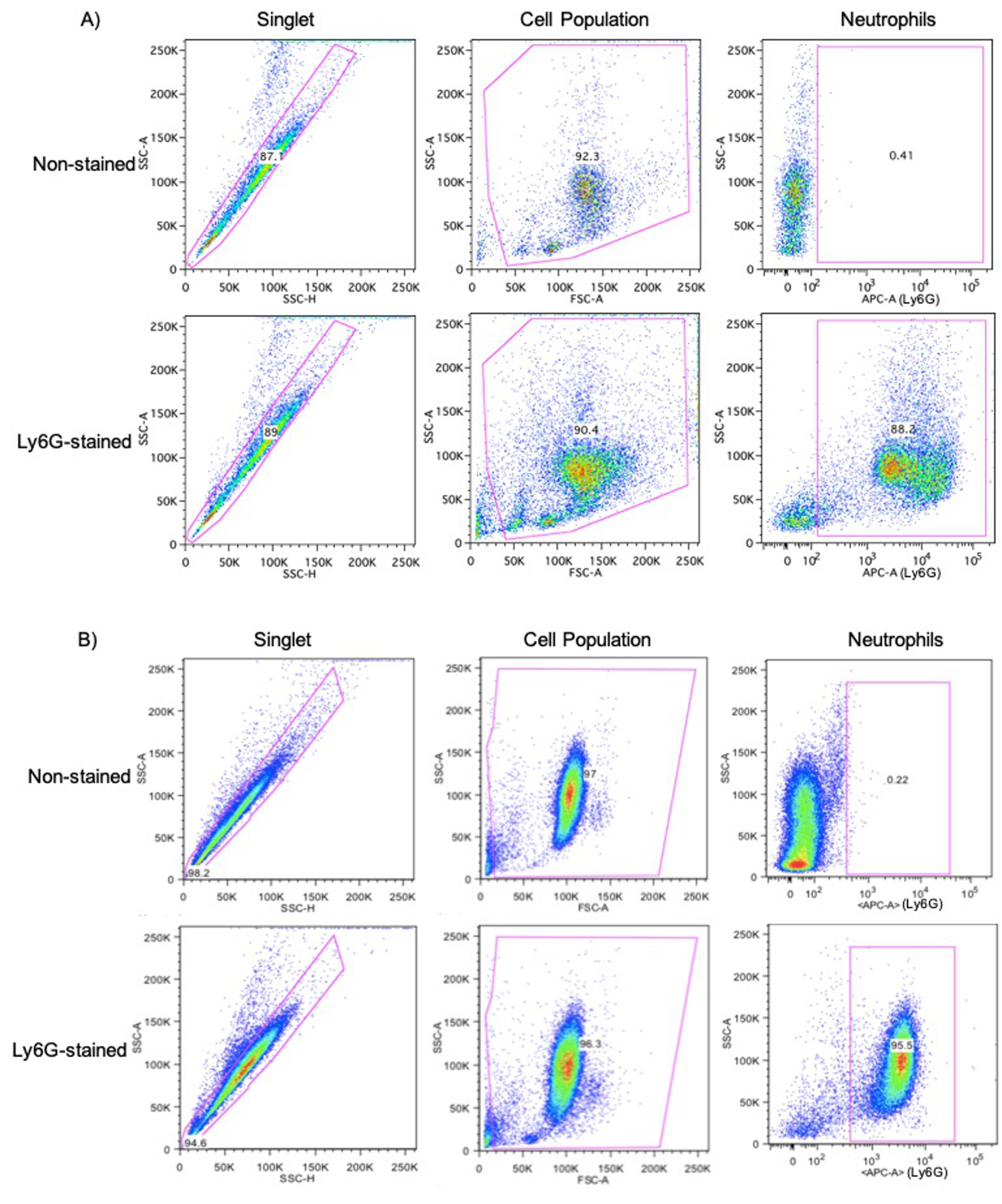

Supplement: Supplementary Figure 1 — Bone marrow neutrophil purity. Bone marrow cells were harvested, and neutrophils were enriched by Ficoll density (A) or using the microbeads anti-Ly-6G ultraPure positive selection kit (Miltenyi Biotec®) (B). After enrichment, cells were stained with anti-Ly6G (APC) antibodies and analyzed by flow cytometry. Neutrophil purity ranged from 85% to 98%. [file Image_1.tif]

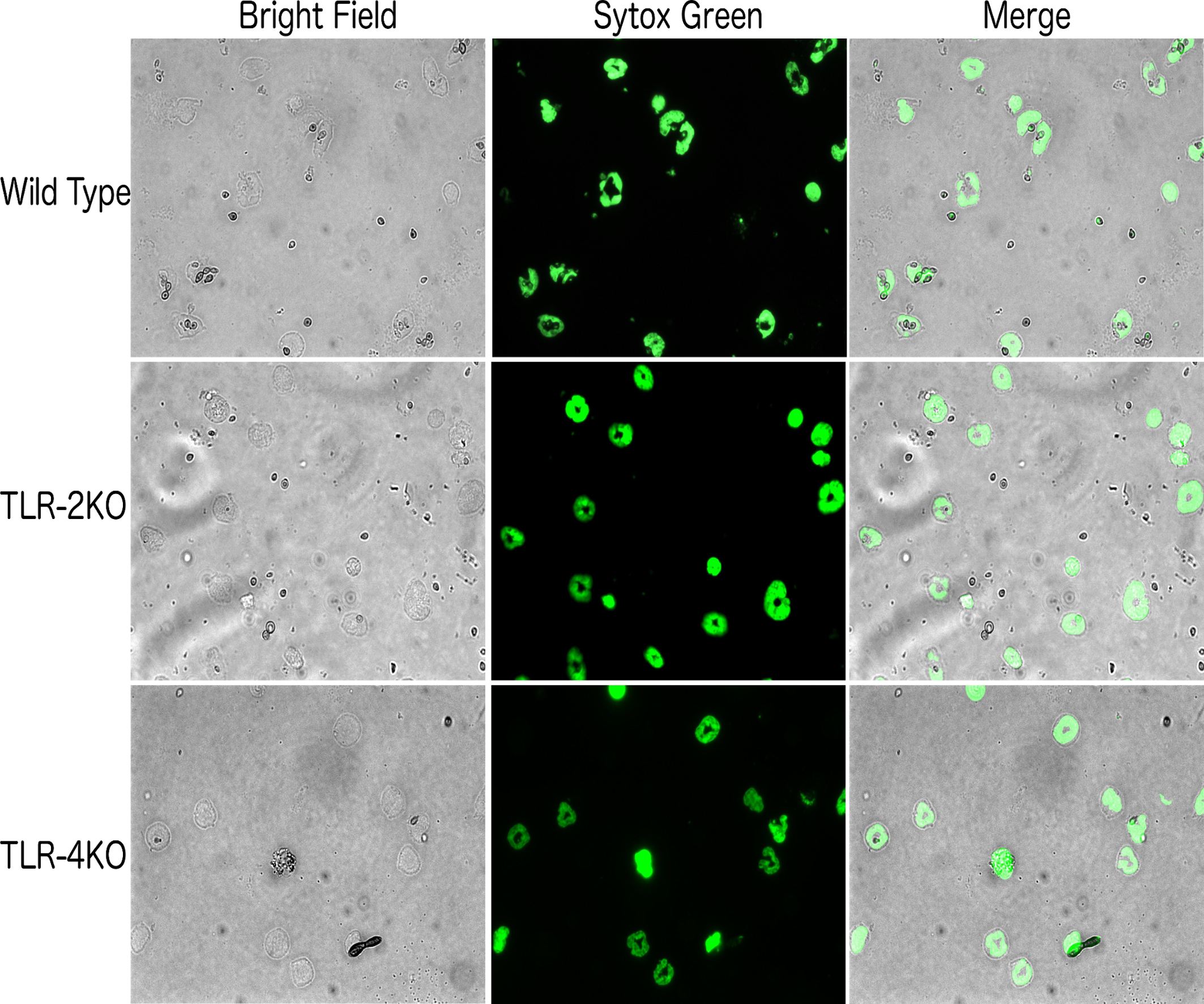

Supplement: Supplementary Figure 2 — Conidia phagocytosis is a TLR-2– and TLR-4–dependent process. WT, TLR-2KO, and TLR-4KO neutrophils were previously purified by Ficoll density layer and then incubated with F. pedrosoi conidia (MOI 1:4) for 120 min. After incubation, the cells were washed and fixed with 4% PFA for 15 min followed by permeabilized with 0.01% PBS-T for 10 min. After washing, the neutrophil nuclei were stained with sytox green, and the slides were mounted using Vecta-Shield® and sealed with nail polish. At least 100 cells were analyzed to calculate the phagocytosis index shown in Figure 1 . [file Image_2.tiff]

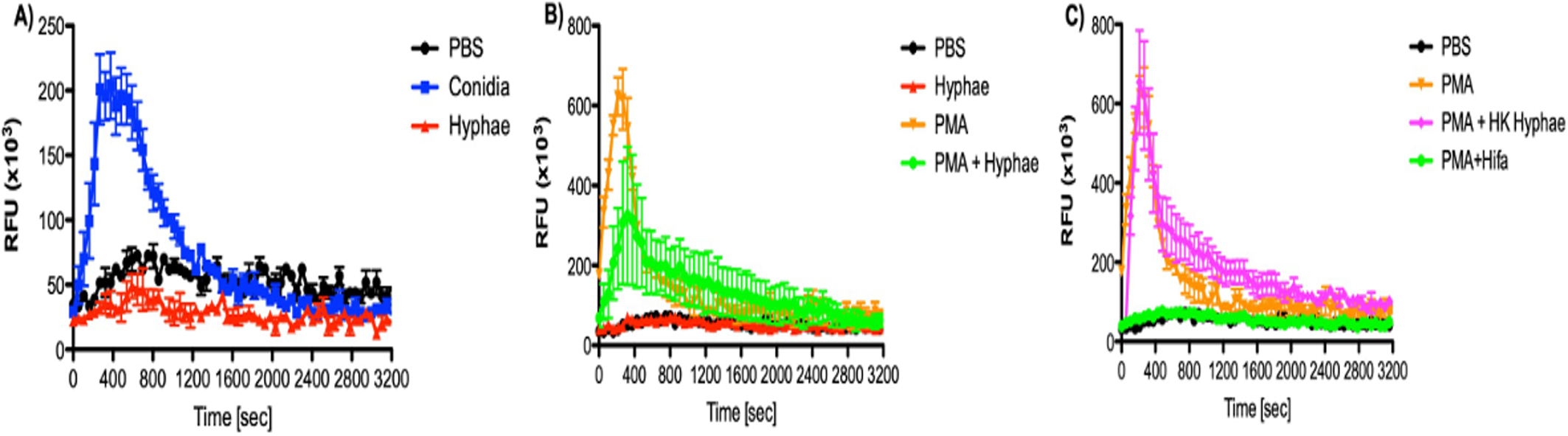

Supplement: Supplementary Figure 3 — Time course of neutrophil ROS production is stimulated by F. pedrosoi conidia and blocked by F. pedrosoi hyphae. (A) WT neutrophils purified by Ficoll were seeded in a 96-well plate in the presence of a luminol reagent and stimulated with F. pedrosoi conidia or hyphae. The ROS production was measured every 2 min to approximately 60 min. As unstimulated control, neutrophils were incubated in the absence of fungi to measure the ROS production during the steady state. The area under the curve was calculated to measure the total ROS production after 60 min. (B) To confirm that hyphae block ROS production, we stimulated the cells with PMA (highly stimulated ROS production) in the presence or absence of hyphae. (C) Using heated-killed (HK) hyphae, we demonstrated that live hyphae blocks, and HK hyphae stimulates ROS production. Data are expressed as mean ± SEM; n = 3. Area under the curve was calculated to quantify the total amount of ROS production. [file Image_3.tiff]

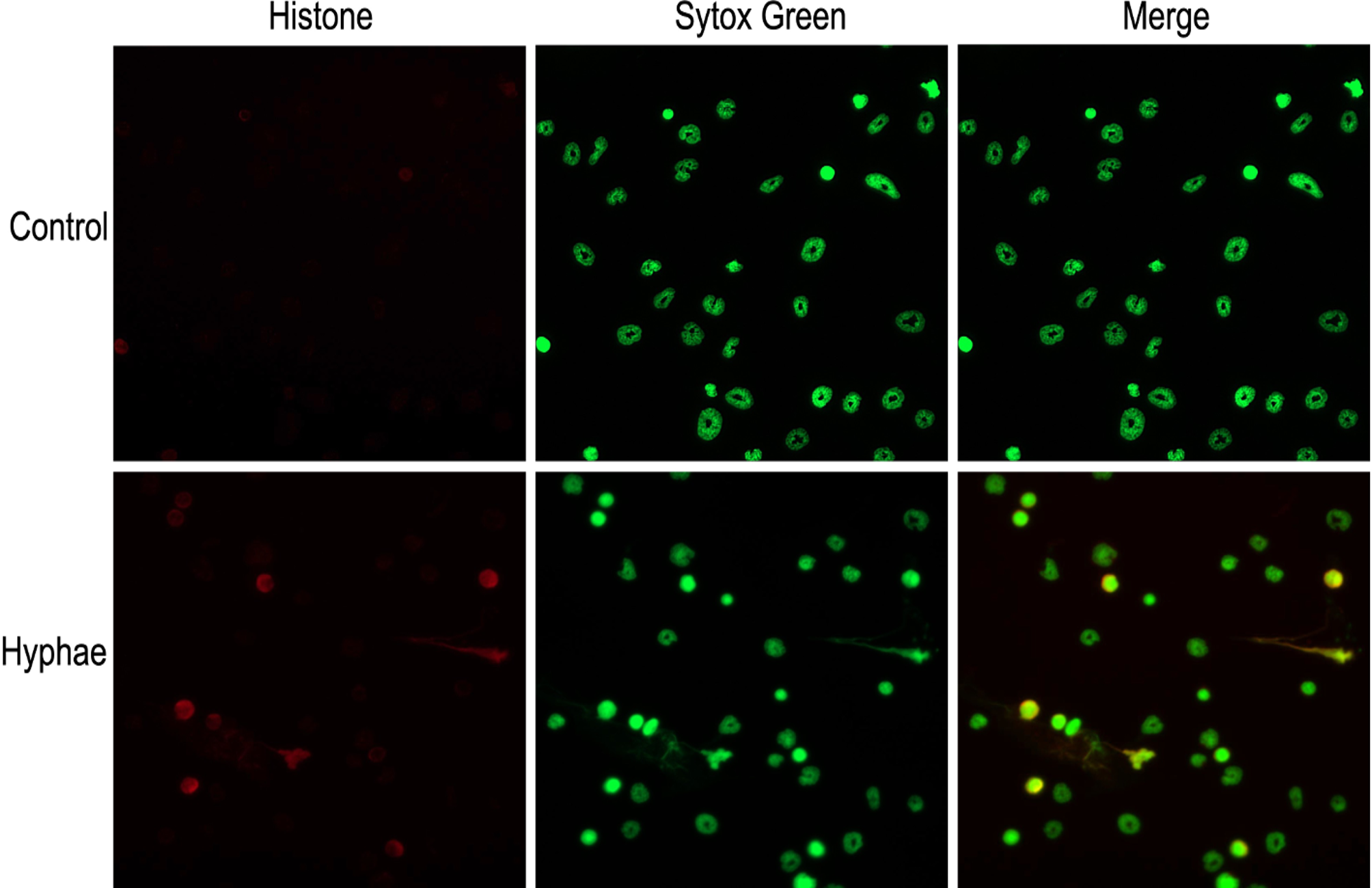

Supplement: Supplementary Figure 4 — Immunofluorescence of NETs stimulated by hyphae of F. pedrosoi. WT neutrophils were resuspended in media (control) or incubated with F. pedrosoi hyphae for 180 min. Afterward, neutrophils were fixed with 4% (v/v) PFA for 15 min, followed by permeabilization with PBS-T for 15 min. Cells were then incubated with antihistone 3 antibody for 1 h followed by incubation with secondary antibody conjugated with alexa-fluor 647 and sytox green dye. After washing, slides were mounted in 5 μL Vecta-Shield® and sealed with nail polish. [file Image_4.tiff]

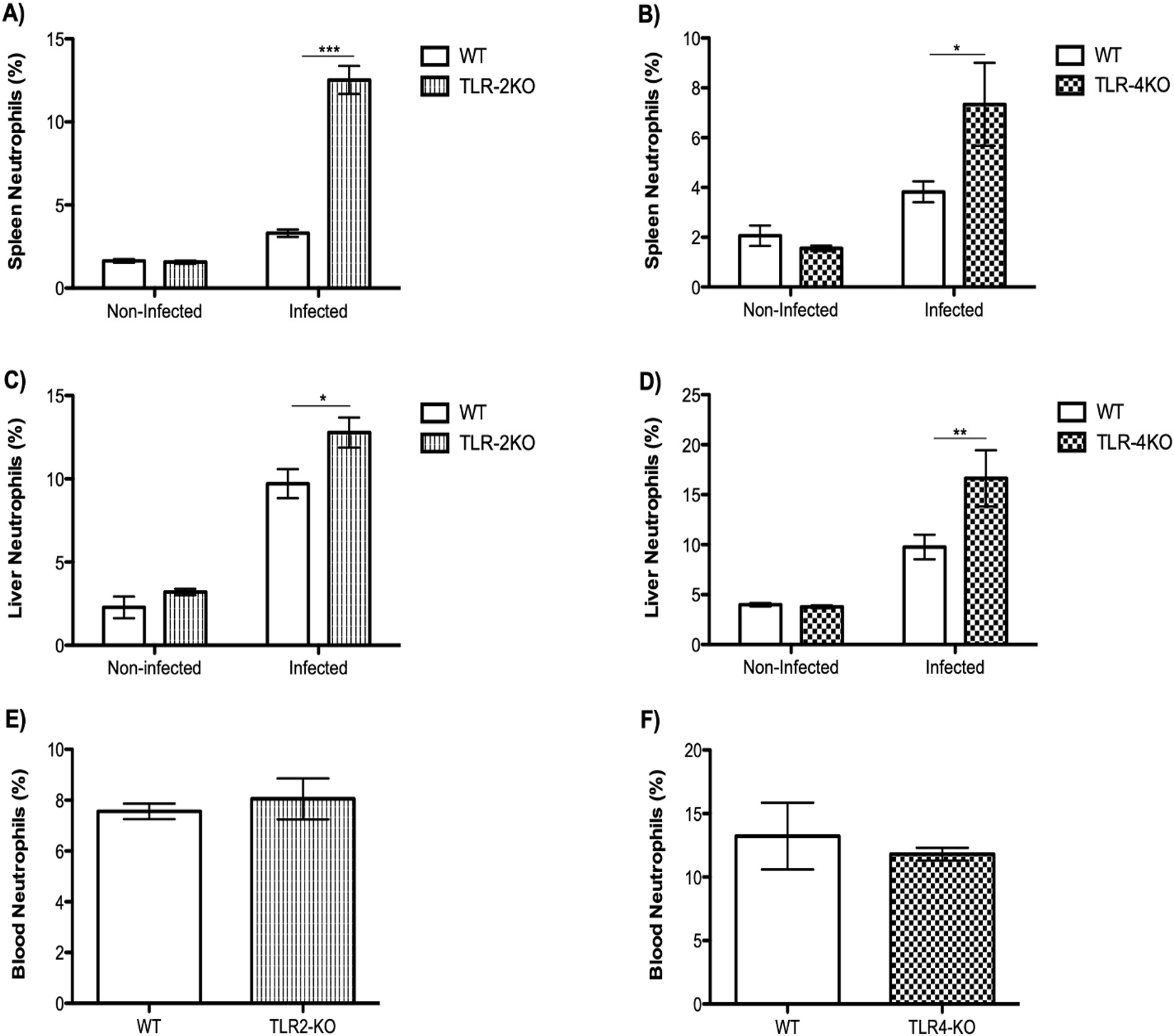

Supplement: Supplementary Figure 5 — Increase of neutrophil population in spleen and liver of TLR-2KO and TLR-4KO animals. Animals were infected i.p. with 5x10^7^ conidia of F. pedrosoi or PBS (noninfected group). After 24 h, the animals were euthanized, and the spleen (A and B) and liver (C and D) were collected for neutrophil analysis by flow cytometry. Peripheral blood from the noninfected group was also collected to verify neutrophil profile in the steady-state condition of WT, TLR-2KO, and TLR-4KO animals (E and F). Data are expressed as mean ± SEM; n = 3–10, two-way ANOVA with Bonferroni’s posttest. *p < 0.05; **p < 0.01; ***p < 0.001. [file Image_5.tiff]

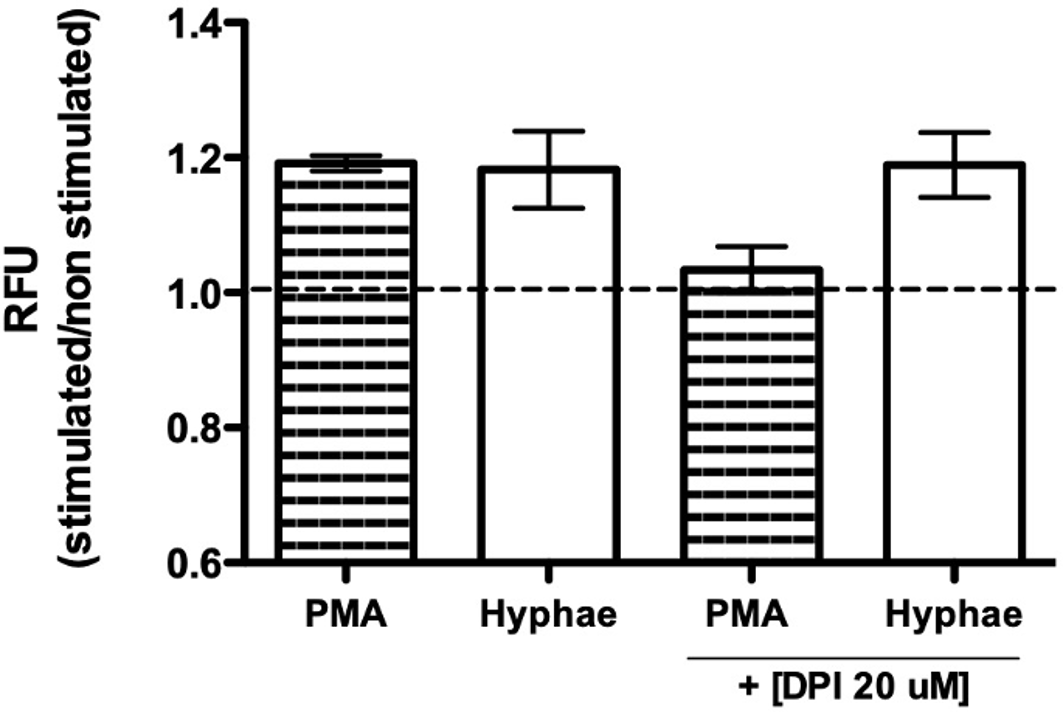

Supplement: Supplementary Figure 6 — NET release in F. pedrosoi hyphae infection is a mechanism independent of NADPH oxidase. WT neutrophils were resuspended in media containing 5 µM sytox green dye in resting condition (dashed lines; negative control) or incubated with PMA or F. pedrosoi hyphae in the presence or absence of 20 μM DPI. After 180 mins, fluorescence was measured and the NETotic index was calculated. In the presence of DPI (20 μM) NET release was deeply inhibited in PMA but not in hyphae-stimulated neutrophils. Data are expressed as mean ± SEM; n=2. [file Image_6.tiff]
